# Optimization of chemically activated carbon derived from malt bagasse for CO₂ adsorption: a simplex-centroid approach

**DOI:** 10.1007/s11356-026-37557-7

**Published:** 2026-02-27

**Authors:** Giovanna C. Carlos, Lucas H. S. Crespo, Pedro H. C. Voloch, Aline A. R. Andrade, Pedro H. V. Ribeiro, Faissal Robbin, Vitor C. Almeida, Lucas Spessato

**Affiliations:** Department of Chemistry, State University of Maringá, Colombo Av. 5790, Maringá, Paraná, Brazil

**Keywords:** Carbon dioxide, Activated carbon, Adsorption, Simplex centroid, Malt bagasse

## Abstract

The intensification of the greenhouse effect has been primarily driven by anthropogenic CO₂ emissions, leading to significant climate change. Among the strategies to mitigate CO₂ emissions from industrial activities, adsorption using activated carbons (ACs) derived from renewable sources stands out as a promising approach. Activated carbons were prepared via chemical activation of malt bagasse, a brewing industry by-product, using NaOH, Na₂C₂O₄, Na₂CO₃, and their mixtures as activating agents. The augmented simplex-centroid method was employed to optimize the process and obtain an activated carbon (ACₒₚ) with high BET surface area. The materials were characterized by proximate analysis, N₂ physisorption, thermogravimetric analysis (TGA), scanning electron microscopy (SEM), Fourier-transform infrared spectroscopy (FTIR), and Raman spectroscopy. CO₂ adsorption experiments were performed at different temperatures to assess performance. The optimized carbon (ACₒₚ) exhibited a well-developed porous structure and high surface area, achieving a CO₂ adsorption capacity of 2.83 mmol g⁻^1^ at 273 K. The results demonstrated that NaOH-activated carbon provides favorable properties for CO₂ capture, confirming the feasibility of malt bagasse as a sustainable precursor for efficient adsorbent materials.

**Supplementary Information:**

The online version contains supplementary material available at 10.1007/s11356-026-37557-7.

## Introduction

The continuous rise in CO_2_ concentration in the atmosphere, mainly attributed to human-driven processes, has become a critical factor in the intensification of global climate disturbances. This phenomenon directly impacts ecological balance, leading to changes such as species extinction, loss of vital ecosystems, scarcity of water resources, and reduction in agricultural production (Weiskopf et al. 2020; Adla et al. 2022). Therefore, recent alternatives have been studied using renewable energy sources and CO_2_ capture and separation techniques, which contribute to reducing greenhouse gases and preserving the environment (Wang et al. 2025; Staudt et al. 2026; Low et al. 2025).

CO_2_ capture and separation techniques involve processes such as electrocatalytic reduction (Deng et al. 2022), absorption with liquid substances (Wang et al. 2024), and adsorption using porous materials (Ye et al. 2025; Sarkar et al. 2025). The adsorption process using highly porous adsorbents has gained prominence as a more energy-efficient strategy. Furthermore, this process is highly effective in removing contaminants, making it a reliable method for purification. Many adsorbents can be engineered to selectively capture specific adsorbates, such as toxic organic compounds and gases, which enhances their usefulness in targeted applications. Additionally, spent adsorbents can be regenerated and reused multiple times, reducing operational costs and minimizing waste production (Fontana et al. 2016; Gkika et al. 2022; Ramar and Balraj 2022).

Adsorption-based process techniques utilize various porous solid materials, including metal–organic frameworks (MOFs) (Abdelkareem et al. 2024; Zelenka et al. 2024), covalent organic frameworks (COFs) (Altaf et al. 2021), porous silica (Li et al. 2024), and carbon-based materials (Zhao et al. 2023; Zelenková et al. 2023). Due to their extensive porosity, chemical and thermal stabilities, high adsorption capacity, and tunable surface chemistry, activated carbons (ACs) have established themselves as multifunctional adsorbents in environmental applications (Almanassra et al. 2021; Zhang et al. 2025). ACs typically exhibit a hierarchical porous structure composed of micropores, mesopores, and macropores. While micropores are primarily responsible for adsorption capacity and mesopores contribute to pore connectivity, macropores play a crucial role as transport channels, facilitating mass transfer and improving the accessibility of micro- and mesopores, particularly in liquid-phase applications (Johnson 2014; Chai et al. 2022). In front of this, ACs are widely used in air purification processes, water and effluent treatment, and also utilized in medical treatments (Singh et al. 2020).

In recent years, special attention has been given to studies to obtain carbonaceous materials from low-cost and widely available renewable sources. In this context, agro-industrial residues have been commonly reported in the literature as promising precursors for preparing ACs. Malt bagasse, a by-product of the beer industry, stands out as a possible precursor to produce ACs due to its considerable contents of cellulose, hemicellulose, and lignin (Neitzel et al. 2023). Given its wide availability, several studies propose its use as a raw material for developing microextraction fibers for removing pesticides in plant samples (Liang et al. 2017), producing advanced materials for energy storage (Duraisamy et al. 2025), and as a precursor for ACs in environmental remediation (Barbosa et al. 2022).

The methods for preparing ACs may involve carbonization and activation processes, which can be either physical or chemical (Ahmad and Azam 2019). The physical activation process utilizes steam, air, and CO_2_ (Yi et al. 2021; Yang et al. 2010). On the other hand, chemical activation involves the use of H_3_PO_4,_ ZnCl_2,_ NaOH, and KOH (Sumangala et al. 2021; Foo and Hameed 2012). In this sense, the literature reports several studies that propose optimizing these parameters using chemometric approaches, with particular emphasis on central composite design (Veza et al. 2023). Researchers often investigate the impact of variation on operational parameters while keeping the amount of chemical reactants constant (Malini et al. 2023). However, it is known that the amount of activating agent is an essential factor in enhancing pore development (Skoczko et al. 2021). Yet, the scientific community frequently disregards this variable, which is becoming increasingly overlooked.

The augmented simplex-centroid (ASC) method is recognized as a valuable tool for investigating how different mixture compositions influence product properties (Presenza et al. 2022; Chraibi et al. 2023). In this context, the ASC method can assist both the scientific community and industry professionals in understanding how the combination of various chemical reactants affects porosity formation and enhances the specific surface area of activated carbon (Spessato et al. 2021). Accordingly, this study aims to produce ACs from malt bagasse through a chemical activation process using a blend of alkaline reagents, with optimization achieved via the ASC method. Furthermore, the optimized ACs were evaluated for their CO_2_ adsorption capacity.

## Materials and methods

### Precursor of AC

The malt bagasse was provided by a brewery in Maringá, Paraná, Brazil. In the laboratory, the material was dried in an oven at 60 °C for 48 h, ground in a knife mill, separated into particles with a size range of 425 to 250 µm, and stored for later studies.

### AC preparation

AC was prepared via a two-step process involving carbonization followed by chemical activation. In the first stage, 20 g of the precursor material was placed into a vertical stainless steel reactor and subjected to pyrolysis in a muffle furnace (EDG3P 7000) at 500 °C for 2.0 h. The process was conducted under a nitrogen atmosphere with a flow rate of 100 cm^3^ min^–1^ and a heating rate of 20 °C min^–1^. After pyrolysis, the system was allowed to cool to room temperature. The resulting carbonized product was then chemically activated using NaOH, Na_2_CO_3_, Na_2_C_2_O_4_, or their combinations, following a fixed mass ratio of 3:1:10 for activating agent, carbonized material, and deionized water, respectively. In each experiment, the components were added to a vertical reactor, magnetically stirred for 1.0 h, and then dried at 150 °C in an oven for 4.0 h. The specific quantities of activating agents were selected based on an augmented simplex-centroid mixture design (as shown in Table 1). The dried samples were subsequently heated to 750 °C for 2.0 h under a nitrogen flow of 100 cm^3^ min^–1^ using the same heating rate as in the carbonization step. After cooling, the activated materials were purified with 0.1 mol L^–1^ HCl solution and deionized water. Finally, the materials were dried at 100 °C for 24 h, stored, and labeled as AC_Y_, where the subscript “Y” corresponds to the experimental run defined in the mixture design.

**Table 1:** Augmented simplex- centroid mixture design and response values

Material	Amount of reagents (g)			*S*_BET_ (m^2^ g ^−1^)
	NaOH (x_1_)	Na_2_CO_3_ (x_2_)	Na_2_C_2_O_4_ (x_3_)	
AC_1_	3.0	0	0	884
AC_2_	0	3.0	0	396
AC_3_	0	0	3.0	368
AC_4_	1.0	2.0	0	358
AC_5_	1.0	0	2.0	540
AC_6_	2.0	1.0	0	405
AC_7_	2.0	0	1.0	318
AC_8_	0	2.0	1.0	381
AC_9_	0	1.0	2.0	476
AC_10_	2.0	0.5	0.5	387
AC_11_	0.5	2.0	0.5	212
AC_12_	0.5	0.5	2.0	427
AC_13_	1.0	1.0	1.0	469

### Experimental design: augmented simplex-centroid method

An augmented simplex-centroid mixture design (Design-Expert® 7.0, Stat-Ease Inc., Minneapolis, MN) was employed to investigate how different mass proportions of NaOH (x_1_), Na_2_CO_3_ (x_2_), and Na_2_C_2_O_4_ (x_3_) influence the specific surface area (*S*_BET_, m^2^ g^–1^) of the resulting activated carbons. A total reactant mass of 3.0 g was maintained for each formulation, leading to the mixture constraint x_1_ + x_2_ + x_3_ = 3.0, or equivalently, a normalized mass fraction where x_1_ + x_2_ + x_3_ = 1.0.

**Fig. 1:**
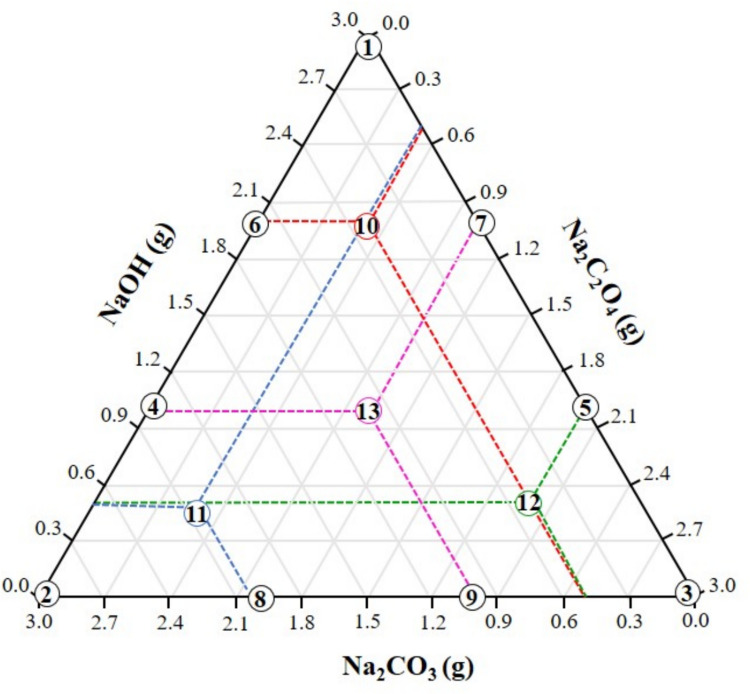
Schematic representation of the augmented simplex-centroid mixture design

The experimental design consisted of 13 distinct formulations, as illustrated in Fig. 1. Experimental points were selected to support the fitting of a higher-order polynomial regression model, which is capable of capturing nonlinear effects and component interactions (Cornell 2011). The design included points at the triangle vertices (pure components), along the edges (binary mixtures), and within the interior (ternary mixtures). The inclusion of additional interior points, characteristic of augmented designs, aimed to increase sampling density, improve model predictability, reduce estimator variance, and enhance the reliability of the response surface (Montgomery 2019).

### Characterizations

The precursor material underwent a centesimal analysis (ASTM-D1762-84) to evaluate its moisture, ash, volatiles, and fixed carbon contents. Using a Shimadzu TGA-50 thermogravimetric analyzer, the thermal stabilities were assessed under a nitrogen flow of 30 mL min^–1^ and a heating rate of 10 ºC min^–1^, starting from room temperature and reaching 800 ºC.

The nitrogen adsorption and desorption isotherms at 77 K were obtained using a QuantaChrome adsorptometer (Nova 1200e). Prior to analysis, the samples were degassed at 150 °C for 12 h under vacuum to remove physisorbed species, such as moisture and volatile compounds. The specific surface area (*S*_BET_) was determined by fitting the linear portion of the Brunauer–Emmett–Teller (BET) equation (Brunauer 1938). The total pore volume (*V*_T_) was defined as the maximum amount of nitrogen adsorbed at a relative pressure of P/P_0_ = 0.99. The micropore volume (V_μ_) was determined using the α-plot method, while the mesopore volume (V_m_) was estimated via the Barret, Joyner, and Halenda (BJH) method (Thommes et al. 2015). The average cylindrical pore diameter (d_P_) was estimated using the pore volume-to-area ratio (4V_T_/*S*_BET_), and the pore distribution was derived using non-local density functional theory (NLDFT) (Landers et al. 2013).

The morphology of both the precursor and the optimized activated carbon was analyzed using scanning electron microscopy (SEM) in a Shimadzu electron microscope (model SS550). To investigate the chemical functional groups, Fourier-transform infrared (FTIR) spectroscopy was employed. The spectra were recorded at a resolution of 4 cm⁻^1^, with 20 scans per minute over a range of 4000 to 400 cm⁻^1^.

Raman spectra were acquired using a Bruker Senterra model dispersive Raman microscope, with an excitation source operating at a wavelength (λ) of 532 nm and a scanning range from 4000 to 400 cm^–1^. The crystallite sizes $({L}_{a})$ were calculated using Eq. (1) (Cançado et al. 2006).

1 $${L}_{a}=\frac{560}{{{E}_{{\boldsymbol{l}}}}^{4}}{\left(\frac{{I}_{D}}{{I}_{G}}\right)}^{-1}$$

where ${{{E}}}_{{{l}}}$ represents the laser excitation energy (eV), and 560 is the proportionality coefficient (which is also related to the transformation from eV to nm).

### Adsorption studies

#### Adsorption equilibrium isotherms at low-pressure conditions

The CO_2_ (99.999%, White Martins) adsorption isotherms were obtained under low pressure (10^−3^, 1.0 bar) using a static volumetric analyzer (QuantaChrome-Nova 1200e). Before the adsorption measurements, the material was degassed at 150 °C for 6.0 h under low pressure (4.0 × 10^−2^ bar). The experiments were realized in a recirculating bath in the temperature range from 273 to 318 K. The adsorption capacity at equilibrium (q_e_, mmol g^− 1^) was calculated using Eq. (2).

2 $${q}_{e}=\frac{1000\rho V}{M}$$

where *ρ* (g cm^−3^) is the density of CO_2_ at STP, *V* (cm^3^ g^−1^) is the adsorbed volume of CO_2_ at STP per gram of dried adsorbent, *M* is the molar mass of CO_2_ (44.01 g mol^−1^), and 1000 is the conversion factor from *mol* to *mmol*.

#### Adsorption selectivity

The analysis was performed at a temperature of 273 K and a pressure range of 0.05 to 1.0 bar using a static volumetric analyzer (QuantaChrome-Nova 1200e). The adsorption selectivity of CO_2_ over N_2_ ($${S}_{{CO}_{2}/{N}_{2}})$$ was calculated using the ideal adsorbed solution theory (IAST) (Eq. 3), commonly employed in predicting adsorption isotherms of mixed gases, based on a set of adsorption isotherms of pure components at the same temperature (Cessford et al. 2012).

3 $${S}_{{CO}_{2}/{N}_{2}}=\frac{{q}_{{CO}_{2}}{P}_{{N}_{2}}}{{q}_{{N}_{2}}{P}_{{CO}_{2}}}$$

where ${q}_{{CO}_{2}}$ and ${q}_{{N}_{2}}$ are the adsorbed amounts (mmol g^−1^) of CO_2_ and N_2_, respectively, and ${P}_{{N}_{2}}$ and ${P}_{{CO}_{2}}$ are the partial pressures (bar) of N_2_ and CO_2_.

#### Enthalpy change of adsorption

The molar enthalpy changes of adsorption, $\Delta {\overline{H} }_{a}$, is a thermodynamic parameter in surface chemistry that depends on the surface coverage (θ). In an isotherm, the pressure P is associated with the volume of adsorbed gas, corresponding to a specific value of θ at a constant temperature T. By analyzing a set of isotherms, it is possible to determine how the pressure of a gas in equilibrium with a solid surface varies with temperature while keeping θ constant. Equation 4 describes the thermodynamic relation between the three mentioned variables, which corresponds to the Clausius–Clapeyron equation.

4 $${\left(\frac{d\;\mathrm{ln}\;p}{dT}\right)}_{\theta }=\frac{-\Delta {\overline{H} }_{a}}{R{T}^{2}}$$

where R is the universal gas constant (8.314 J K^−1^ mol^−1^).

### Adsorption equilibrium isotherms at high-pressure conditions

High-pressure CO₂ adsorption experiments (up to 20 bar) were performed using a magnetic suspension balance (MSB) (Rubotherm® Isosorp™ HyGrA, Bochum, Germany). Prior to each experiment, 0.05 g of the prepared materials were subjected to a thermal pre-treatment at 150 °C for 12 h under ultra-high vacuum (10⁻^6^ bar) to remove residual moisture and unblock the pore structure. Data analysis was conducted following the methodology proposed by Dreisbach et al. (2002). Specifically, the equilibrium CO_2_ adsorption capacity (${q}_{e}$, mmol g⁻^1^) at elevated pressures was calculated using Eqs. (5–7).

5 $${q}_{e}=\frac{{m}_{a}}{{m}_{s}}\left(\frac{1000}{M}\right)$$

6 $${m}_{a}=\frac{\Omega -\frac{\rho }{{\rho }_{s}}{m}_{s}}{1-\frac{\rho }{{\rho }_{a}}}$$

7 $$\Omega =\Delta m-{m}_{s}-{m}_{sc}+{V}_{sc}\rho$$

where 1000 is the conversion factor from *mol* to *mmol*, *m*_*a*_ is the absolute adsorbed mass (g); *m*_*s*_ is the dried mass (g); *M* is the CO_2_ molar mass (44.01 g mol^−1^); $\Omega$ is the reduced adsorbed quantity (g); $\Delta m$ is the experimental mass change measured by the MSB (g); *m*_*sc*_ and *V*_*sc*_ are the mass (g) and volume (cm^3^) of the sample container, respectively; $\rho$_a_ is the density of the adsorbed phase (g cm^−3^); $\rho$_s_ is the skeletal density of the solid material (g cm^−3^), determined by He pycnometry (99.999%, White Martins); and $\rho$ is the gas density (g cm^−3^). All experiments were conducted at 25 °C, and the gas density values were calculated using the Span-Wagner equation of state, implemented in the NIST Reference Fluid Thermodynamic and Transport Properties Database (REFPROP) software.

## Results and discussions

### Centesimal analysis

The proximate analysis of the precursor provided important insights into its characteristics. The results indicate that malt bagasse is a suitable precursor to produce activated carbons, exhibiting a low ash content of 2.19 ± 0.12% and a high fixed carbon content of 26.34 ± 0.10%. This high fixed carbon content is directly related to charcoal yield, mainly due to the contribution of lignin, which, owing to its complex aromatic structure and high thermal resistance, constitutes the main source of residual carbon after pyrolysis (Jjagwe et al. 2021). Furthermore, the high volatile matter content, measured at 71.47 ± 0.03%, reveals that malt bagasse is predominantly composed of thermally unstable compounds such as carbohydrates, proteins, lipids, as well as hemicellulose and cellulose, which also contribute significantly to this fraction, as they typically degrade within the 200–400 °C range (Xu et al. 2024).

### Experimental design–augmented centroid simplex method

#### Model building and analysis of variance (ANOVA)

The augmented simplex-centroid mixture design incorporates three components to achieve different proportions of chemicals. The selection of reagents NaOH, Na_2_CO_3_, and Na_2_C_2_O_4_ is based on their widespread use as the most common chemical activating agents in the production of activated carbons (Ilnicka and Lukaszewicz 2015; Rodríguez-Mosqueda et al. 2018; Romero-Hernandez et al. 2024; Premchand et al. 2024). These alkali-based compounds are recognized for their effectiveness in enhancing porosity and surface area, which are crucial properties for applications such as adsorption and catalysis (Petrovic et al. 2022). Their ability to facilitate the development of well-defined pore structures makes them fundamental choices in the field of chemical activation, ensuring high-performance activated carbons tailored for specific industrial and environmental applications.

Table 1 displays the design matrix and the experimental *S*_BET_ values (m^2^ g⁻^1^) for each test. The results reveal that *S*_BET_ values varied from 212 to 884 m^2^ g⁻^1^, clearly indicating that both the chemical composition and the chemical nature of the mixtures significantly influence pore development. Several mathematical models were applied to experimental data, as shown in Table S1. Notably, the cubic model was the only one to yield a satisfactory fit to the *S*_BET_ values, achieving the highest *R*^2^ and adjusted *R*^2^, as well as the lowest standard deviation.

To numerically assess the quality of the fitted cubic model, an analysis of variance (ANOVA) was conducted, with results shown in Table S2. Fisher’s distribution (*F*-value) and the corresponding probability value (*p*-value) were used at a 95% confidence level to evaluate the significance of the model and its interaction terms. For the model and its interaction terms to be considered significant, it must show a *p*-value < 0.05 and *F*_calculated_ > *F*_tabulated_, given its degrees of freedom. Table S2 reveals that the model *F*_calculated_ = 4.86, exceeding the critical tabulated value *F*_9,16_ = 2.54, confirming the significance of the model. Additionally, the model produced a *p*-value of 0.0030, which is below the 0.05 threshold required for statistical significance.

The significance of the interaction terms was similarly examined. The linear terms exhibited a *p*-value of 0.0054 and a *F*_calculated_ value of 7.35, which is greater than the critical value of F_2,16_ = 3.63, thereby demonstrating statistical significance. Consequently, the pure components NaOH, Na_2_CO_3_, and Na_2_C_2_O_4_ substantially influence the development of a porous structure within the carbonaceous matrix. The quadratic interaction terms ${\mathrm{x}}_{1}{\mathrm{x}}_{2}$ (NaOH-Na_2_CO_3_) and ${\mathrm{x}}_{1}{\mathrm{x}}_{3}$ (NaOH-Na_2_C_2_O_4_) and the cubic interaction term ${\mathrm{x}}_{1}{\mathrm{x}}_{3}{(\mathrm{x}}_{1}-{\mathrm{x}}_{3})$ presented statistical significance with *p*-values lower than 0.05, and *F*_calculated_ values greater than the tabulated F_1,16_ = 4.49, indicating that the combination of reagents has an important influence in the formation of porosity. On the other hand, the terms ${\mathrm{x}}_{2}{\mathrm{x}}_{3}$ (Na_2_CO_3_-Na_2_C_2_O_4_), ${\mathrm{x}}_{1}{\mathrm{x}}_{2}{\mathrm{x}}_{3}$ (NaOH-Na_2_CO_3_-Na_2_C_2_O_4_), ${\mathrm{x}}_{1}{\mathrm{x}}_{2}{(\mathrm{x}}_{1}-{\mathrm{x}}_{2})$ and ${\mathrm{x}}_{2}{\mathrm{x}}_{3}{(\mathrm{x}}_{2}-{\mathrm{x}}_{3})$ are considered statistically insignificant terms once presented *p*-values greater than 0.05 and *F*_calculated_ values lower than the tabulated F_1.16_. Therefore, these specific combinations of reagents do not significantly affect the development of a porous structure within the carbonaceous matrix. As verified by the treatment using ANOVA, the final equation to predict the *S*_BET_ response, using only the significant terms, is given by Eq. 8.

8 $${\mathrm{S}}_{\mathrm{BET}}=290.27{\mathrm{x}}_{1}+130.20{\mathrm{x}}_{2}+120.50{\mathrm{x}}_{3}-119.75{\mathrm{x}}_{1}{\mathrm{x}}_{2}-95.11{\mathrm{x}}_{1}{\mathrm{x}}_{3} -78.16{\mathrm{x}}_{1}{\mathrm{x}}_{3}({\mathrm{x}}_{1}-{\mathrm{x}}_{3})$$

The regression model shows that the linear coefficients associated with x_1_ (NaOH), x_2_ (Na_2_CO_3_), and x_3_ (Na_2_C_2_O_4_) are positive and of high magnitude, indicating that an increase in these factors tends to produce a proportional enhancement in *S*_BET_. It is important to highlight that the increase in *S*_BET_ is more prominent by an increase in x_1_, followed by x_2_ and then x_3_. Conversely, the quadratic interaction terms ${\mathrm{x}}_{1}{\mathrm{x}}_{2}$ and ${\mathrm{x}}_{1}{\mathrm{x}}_{3}$, as well as the significant cubic contribution, exhibit negative coefficients. This behavior suggests the existence of a concave response surface in these directions, where excessive increases in the factor levels lead to diminishing returns and, ultimately, a decrease in the response. Such trends are consistent with the presence of optimal factor ranges, beyond which adverse effects, such as particle sintering, surface blockage by residual species, or collapse of porous structures, can reduce the available surface area for adsorption, thereby lowering *S*_BET_.

**Fig. 2:**
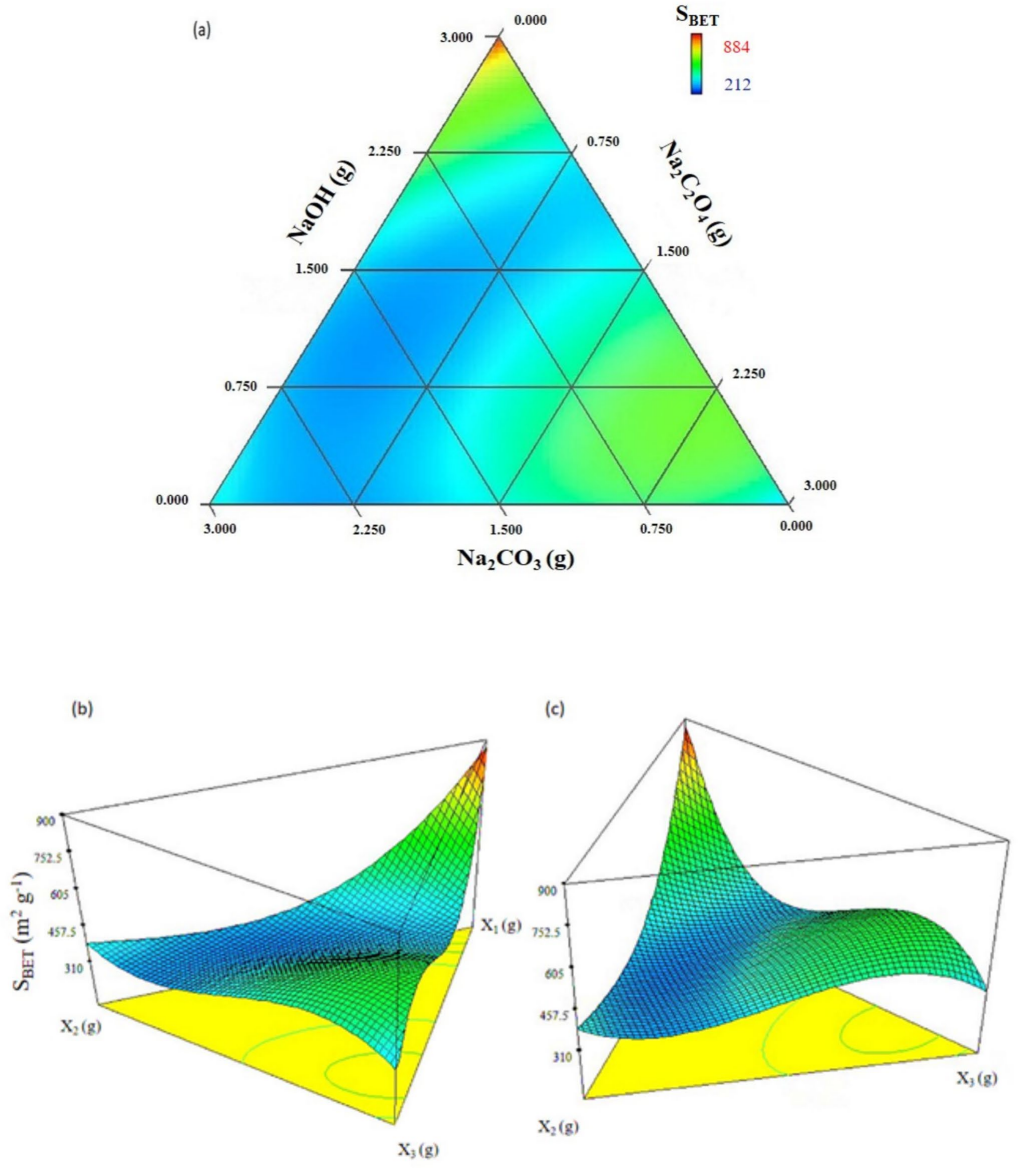
**a** Contour plot and **b**, **c** 3D surface of the experimental values for the area

The coefficient of determination (*R*^2^) for the model was 0.7323, indicating that the model explains 73.23% of the variation around the mean. This leaves 26.77% as residuals, which are distributed according to the graph presented in Fig. S1. The graphical analysis of the residuals showed that the results are homoscedastic, meaning the variance of the errors remains constant throughout the entire range studied. Figure 2 illustrates the response projection for specific surface area (*S*_BET_, m^2^ g⁻^1^) obtained from the experimental design, presented as a contour plot and 3D surface graphs.

Statistical analysis indicated that the interaction terms ${\mathrm{x}}_{1}{\mathrm{x}}_{2}$ (NaOH-Na_2_CO_3_), ${\mathrm{x}}_{1}{\mathrm{x}}_{3}$ (NaOH-Na_2_C_2_O_4_), and the cubic term ${\mathrm{x}}_{1}{\mathrm{x}}_{3}({\mathrm{x}}_{1}-{\mathrm{x}}_{3})$ exhibited negative coefficients in the model, showing that these combinations decrease the specific surface area (*S*_BET_) compared to the use of pure NaOH. This trend can be explained by the thermal sequence and activation mechanisms of each reagent, as well as by their chemical interactions.

NaOH initiates activation processes at relatively low temperatures (~ 300 °C), at which it melts and progressively interacts with the carbonaceous matrix, promoting dehydration, structural rearrangements, and the early evolution of gaseous species (H_2_, CO, and CO_2_). These phenomena contribute to the initial development of microporosity and the formation of fine pore connections within the carbon network. When combined with Na_2_CO_3_ (x_1_x_2_), whose significant thermal decomposition occurs only at higher temperatures (~ 500 °C) (Ballirano 2011), the overall reactivity of the activating system is effectively diluted, leading to a delayed and less aggressive activation behavior. This likely reduces the density of reactive sites in the temperature range most favorable to micropore nucleation. At higher temperatures, however, the process shifts toward gasification and the widening of pre-existing pores, which tends to compromise microporosity and, consequently, reduces *S*_BET_.

Thermal behavior of Na_2_CO_3_ shows distinct polymorphic transitions beginning around 247 °C, indicating structural instabilities prior to any chemical decomposition (Ballirano 2011), indicating that Na_2_CO_3_ remains largely inert during the initial activation window dominated by NaOH, which engages at much lower temperatures contributing to effective micropore formation. Notably, classical thermochemical data (Barin 1993) establish that Na_2_CO_3_ does not decompose to Na_2_O and CO_2_ until temperatures approach or exceed its melting point (~ 850 °C), reinforcing that decomposition is not a factor during early or even intermediate stages of activation.

Similarly, the NaOH-Na_2_C_2_O_4_ interaction (${\mathrm{x}}_{1}{\mathrm{x}}_{3}$) leads to thermal desynchronization. Na_2_C_2_O_4_ decomposes only between 750 and 800 °C (Chaiyo et al. 2012), releasing CO and CO_2_ at a late stage when the structure previously activated by NaOH is more susceptible to coalescence and local collapse of carbon walls. This late gas release tends to enlarge micropores and reduce surface area, an effect further aggravated by the conversion of oxalate into sodium carbonate or oxide, which may also react with residual NaOH and alter the wettability and homogeneous penetration of the activating medium.

The negative cubic term ${\mathrm{x}}_{1}{\mathrm{x}}_{3}({\mathrm{x}}_{1}-{\mathrm{x}}_{3})$ captures the strongly nonlinear behavior of this combination: a small reduction in the NaOH fraction accompanied by increases in Na_2_C_2_O_4_ quickly shifts the system from an efficient activation regime (dominated by early-formed micropores) to a late-stage gasifying regime, with higher mesoporosity and lower *S*_BET_. Thus, the model’s statistics directly reflect the physicochemical process: the highest surface area is obtained with pure NaOH at a 3:1 ratio, whereas the studied mixtures reduce microporosity due to strong base dilution, desynchronization of thermal activation windows, and in situ carbonation effects.

The activation mechanism can be understood by considering the thermal sequence, phase behavior, and chemical reactivity of each activating agent, as well as their interactions with the carbon matrix. As already discussed, NaOH exhibits the highest activation efficiency due to its relatively low melting point and high reactivity with carbon at elevated temperatures. Upon melting, NaOH penetrates the carbon framework and reacts directly with the carbon matrix, producing metallic sodium at high temperature and gaseous species (H_2_ and CO/CO_2_), which promotes extensive carbon gasification and pore development (Reaction R1). The intercalation of metallic sodium further expands the carbon lattice, contributing to the formation of a highly developed microporous structure.

In contrast, Na_2_CO_3_ acts as a weaker activating agent. Its activation pathway involves thermal decomposition into Na_2_O and CO_2_ (Reaction *R*^2^), followed by secondary gasification reactions between CO_2_ and carbon (Reaction R3). At higher temperatures, Na_2_CO_3_ and Na_2_O can also react directly with carbon to form metallic sodium and CO (Reactions R4 and R5), although these reactions occur to a lesser extent compared to NaOH. As a result, the pore development promoted by Na_2_CO_3_ is significantly less pronounced.

Sodium oxalate (Na_2_C_2_O_4_) does not directly activate the carbon framework. Instead, it undergoes thermal decomposition, yielding Na_2_CO_3_ and gaseous products (Reaction R6). Consequently, its contribution to pore formation is indirect and occurs mainly through the subsequent reactions of the generated Na_2_CO_3_, explaining the limited enhancement of specific surface area observed experimentally (Feng et al. 2025).

Therefore, the combined interpretation of thermal properties (melting and decomposition temperatures), reaction pathways, and gas–solid interactions provides a consistent mechanistic explanation for the superior performance of NaOH and the comparatively weaker activation effects of Na_2_CO_3_ and Na_2_C_2_O_4_, in agreement with the statistical trends revealed by the ANOVA analysis.

**Table:** 

*Activation by KOH*	
6NaOH + 2C→2Na + 3H_2_ + 2Na_2_CO_3_	(R1)
*Activation by Na*_*2*_*CO*_*3*_	
Na_2_CO_3_ →Na_2_O + CO_2_	(R2)
CO_2_ + C→2CO	(R3)
Na_2_CO_3_ + 2 C→2Na + 3CO	(R4)
Na_2_O + C→2Na + CO	(R5)
*Activation by Na*_*2*_*C*_*2*_*O*_*4*_	
2Na_2_C_2_O_4_ →2Na_2_CO_3_ + C + CO_2_	(R6)

#### Optimization of AC preparation conditions

The optimization condition determined by the Design Expert® software aimed to maximize the specific surface area (*S*_BET_) of the activated carbon (AC) within the experimental range studied. The optimization indicated that an impregnation ratio of 3:1 with the NaOH reagent would provide the highest predicted surface area, estimated at 870 m^2^ g^−1^. For the evaluated condition, a desirability index of 0.968 was obtained (Fig. S2), a value approaching the ideal maximum of 1.0. In this methodology, each response is transformed into an individual desirability function ranging from 0 (unacceptable) to 1.0 (optimal) (Srikar and Rani 2020). Therefore, a value of 0.968 indicates that the chosen conditions nearly achieve the desired targets, underscoring the excellence of the experimental parameters and validating the optimization process carried out with the Design Expert® software. The experimental results confirmed the prediction, achieving an *S*_BET_ of 886 m^2^ g^−1^, demonstrating good agreement between theoretical and experimental values. These results validate the effectiveness of the predictive model used. Given this performance, the material obtained under these conditions, referred to as AC_op_, was selected for further characterization and adsorption studies.

### Thermogravimetric analysis

**Fig. 3:**
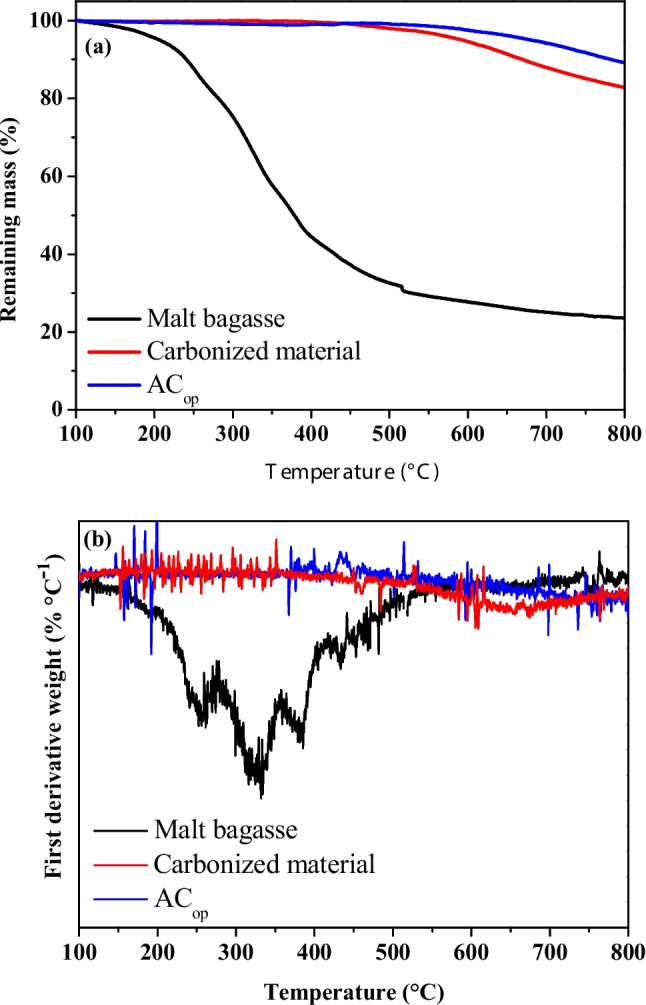
(**a**) Thermogravimetric (TG) and (**b**) derivative thermogravimetric (DTG) curves of the malt bagasse, carbonized material, and AC_op_

The thermal degradation behavior of malt bagasse, carbonized material, and AC_op_ were investigated by thermogravimetric (TG) and its first derivative (DTG) curves, as shown in Fig. 3. The TG and DTG profiles clearly demonstrate the progressive increase in thermal stability from the raw biomass to the carbonized material and AC_op_, reflecting the structural transformations induced by thermal treatments.

The malt bagasse exhibits a typical multistep thermal degradation profile characteristic of lignocellulosic biomass. An initial mass loss below approximately 150 °C is attributed to the removal of physically adsorbed moisture, followed by a major degradation step between 200 and 400 °C, which might be attributed to the overlapping thermal decomposition of hemicellulose and cellulose (Nadolny et al. 2020). The most intense degradation occurs in this temperature range, leading to a sharp decrease in the remaining mass from 95.6 to 44.4%. Above 400 °C, a slower mass loss associated with lignin decomposition and carbonaceous matrix formation is observed (Apaydın and Mutlu 2023; Chen et al. 2022), resulting in a residual mass of 23% at 800 °C, which can be attributed to fixed carbon and inorganic ash content.

In contrast, the carbonized material shows an improved thermal stability. Only minor mass losses are observed up to approximately 500 °C, indicating that most thermally labile organic components were removed during the carbonization process. At higher temperatures, a gradual mass loss occurs, mainly associated with the decomposition of residual oxygen-containing functional groups and further structural rearrangement of the carbon matrix (Zhang et al. 2024). At 800 °C, the carbonized material retains a residual mass equal to 82.9%, confirming the formation of a more stable carbonaceous structure.

The AC_op_ exhibits the highest thermal stability among the three materials. The TG curve shows the highest residual mass over the entire temperature range (89.2% at 800 °C), with a slow decrease at higher temperatures. This behavior reflects the predominance of condensed aromatic carbon structures and a high content of fixed carbon. The enhanced thermal resistance of the AC_op_ can be attributed to the activation process, which promotes the elimination of unstable carbon fractions and the development of a more ordered and thermally robust carbon framework (Choi et al. 2023).

The DTG curves (Fig. 3b) provide further insight into the degradation mechanisms. The malt bagasse displays three pronounced DTG peaks between 250 and 380 °C, corresponding to the maximum decomposition rates of hemicellulose and cellulose. In contrast, both the carbonized material and the AC_op_ exhibit low-intensity DTG signals without distinct peaks, indicating the absence of well-defined decomposition events. The slightly higher DTG signal observed for the carbonized material at 650 °C suggests a lower thermal resistance compared to the AC_op_, which shows the lowest degradation rates throughout the analysis.

### Textural characterization

**Fig. 4:**
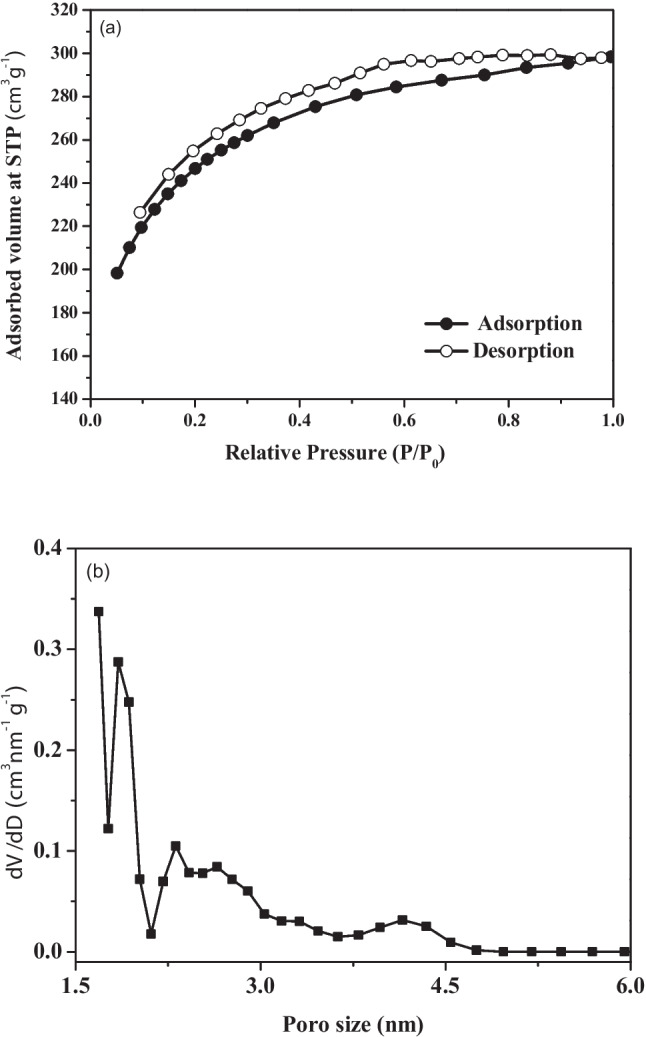
**a** Adsorption and desorption isotherms and **b** pore size distribution of AC_op_

N_2_ adsorption and desorption isotherms are shown in Fig.4a. The adsorption curve shows that as the relative pressure increases, the amount of N_2_ adsorbed by the material rises. In contrast, the desorption curve displays different behavior, leading to hysteresis between the adsorption and desorption processes. This phenomenon is typical of microporous-mesoporous materials (Thommes et al. 2015). According to the IUPAC classification, the isotherm profile corresponds to type I(b), indicating the presence of wider micropores and narrow mesopores, with widths of less than 2.5 nm, and type H4 hysteresis related to micropore filling. Figure 4b illustrates the pore diameter distribution of AC_op_. As observed, the maximum distribution occurs in pores with diameters up to 2.0 nm, indicating a substantial amount of micropores.

The results demonstrated that NaOH acted effectively as an activating agent in the development of porosity. This increase can be attributed to degradation reactions induced by the presence of NaOH, which promote the formation of graphitic carbon layers. As a consequence of these structural modifications, gases such as H_2_, CO_2_, and CO are generated, directly contributing to the enhancement of the material’s porosity (Heidarinejad et al. 2020). Moreover, the initial reaction between NaOH and carbon occurs at a relatively low temperature, around 300 °C (Lillo-Ródenas et al. 2003), enhancing the efficiency of subsequent thermal reactions and resulting in the release of a greater amount of gases and metallic sodium (Aloud et al. 2023). The possible reactions involved are presented in reaction R1–R5 (Alfatah et al. 2021).

The pore distribution shows that the materials have more micropores than mesopores, confirming the classification of the adsorption/desorption isotherm for microporous materials. Table 2 summarizes the textural properties of AC_op_ and compares them with literature reports on NaOH-activated carbons, including key preparation and activation parameters. The obtained values are in good agreement with those reported in previous studies, confirming the reliability of NaOH-based chemical activation for tailoring carbon porosity.

**Table 2:** Preparation conditions and textural properties of NaOH-activated carbons from different precursors, including AC_op_

Precursor	^*^CM:NaOH ratio (w:w)	Temperature (°C)	N_2_ flow (cm^3^ min^−1^)	Time (h)	Heating rate (°C min^−1^)	*S*_BET_(m^2^ g ^−1^)	*V*_T_ (cm^3^ g^−1^)	*V*μ (cm^3^ g^− 1^)	*V*_M_ (cm^3^ g^−1^)	*D*_p_ (nm)	Reference
Malt bagasse	1:3	750	100	2.0	20	884	0.460	0.420	0.040	2.05	This work
Saturated AC	6:2	700	10	1.0	5.0	1105	0.777	nr	nr	2.81	(Wu et al. 2025)
Saturated AC	6:8	700	10	1.0	5.0	1959	1.263	nr	nr	2.58	(Wu et al. 2025)
Macroalgae	1:2	800	150	1.5	10	1238	0.682	nr	nr	2.20	(Wei et al. 2022)
Rattan	1:3	600	150	1.0	10	1135	0.600	0.170	0.440	3.55	(Islam et al. 2017)
Rice husk	1:3	800	nr	2.0	5.0	1857	1.389	0.432	nr	1.50	(Zhang et al. 2014)
Rice husk	1:3	800	nr	2.0	5.0	933	0.398	0.346	nr	1.71	(Song et al. 2012)
Coffee Gounds	1:2	450	nr	1.0	15	664	0.468	nr	nr	0.88	(Qisse et al. 2022)
Coconut Residue	1:1	600	300	1.0	nr	190	0.120	0.08	0.040	nr	(Yahya et al. 2016)
Coconut Residue	1:2	600	300	1.0	nr	564	0.320	0.260	0.060	nr	(Yahya et al. 2016)
Coconut Residue	1:3	600	300	1.0	nr	1200	0.680	0.520	0.160	nr	(Yahya et al. 2016)
Sugarcane bagasse	1:4	850	nr	1.5	nr	1149	1.730	0.080	nr	6.02	(Guo et al. 2020)
*Camellia oleifera*	1:4	700	nr	2.0	nr	512	0.356	0.192	0.160	2.78	(Quan et al. 2023)
Cellulose	1:4	600	100	2.0	10	1334	0.610	0.340	0.270	nr	(Taher et al. 2023)

^*^Carbonized material; *nr* not reported

### Morphological characterization

**Fig. 5:**
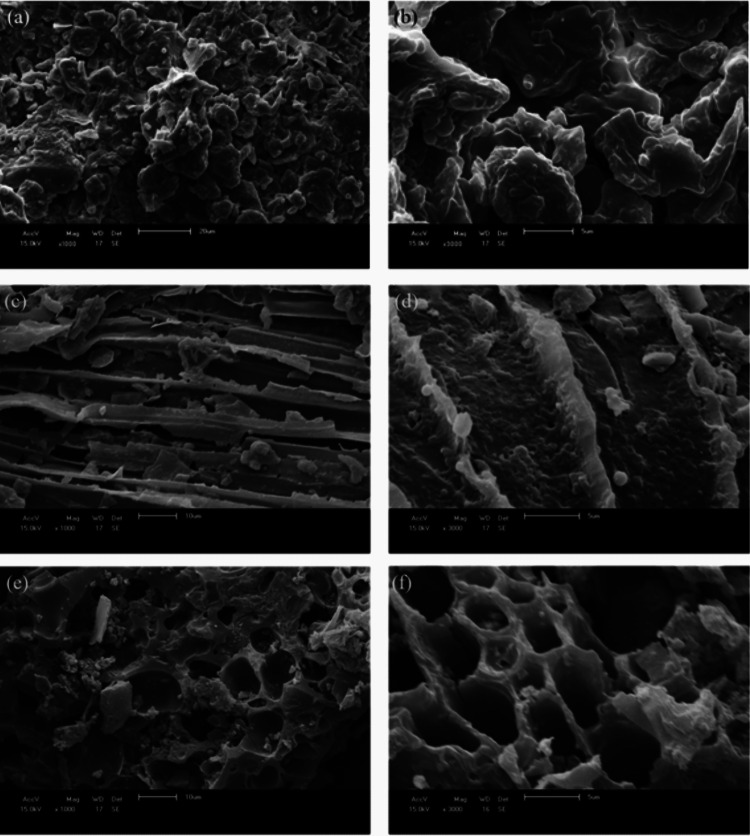
SEM images of malt bagasse (**a**, **b**), carbonized material (**c**, **d**), and AC_op_ (**e**, **f**)

SEM images of malt bagasse (Fig. 5a–b) show fibrous walls with relatively smooth surfaces and partially occluded lumens, indicative of the native plant microstructure. After carbonization (Fig. 5c–d), the structure partially collapses and displays cracks and fissures; pore openings remain limited, consistent with incomplete removal of volatiles and preservation of blocked channels. In the activated carbon (Fig. 5e–f), NaOH-driven intercalation and chemical etching generate a fragmented surface with abundant cavities and interconnected channels. Removal of inorganic residues during washing exposes a hierarchical network in which larger openings connect microporous domains, in agreement with the higher *S*_BET_.

### Fourier transform infrared spectroscopy (FTIR)

**Fig. 6:**
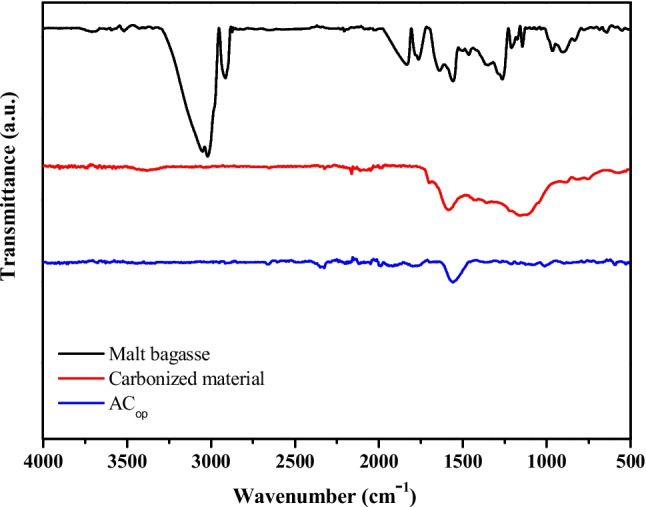
FTIR–ATR spectrum of malt bagasse, carbonized material, and AC_op_

The FTIR-ATR spectra (Fig. 6) provide clear evidence of the chemical transformations that occur during the conversion of malt bagasse into carbonized material and subsequently into activated carbon (AC_op_). For the raw malt bagasse (black spectrum), several intense absorption bands are observed. The broad band around 3300–3400 cm^−1^ corresponds to O–H stretching vibrations from hydroxyl groups (alcohols, phenols, and adsorbed water) (Fontana et al. 2016). The signals at approximately 2920 and 2850 cm^−1^ are attributed to C–H stretching vibrations of aliphatic –CH_2_ groups (Liñán-Montes et al. 2014). The strong band near 1730 cm^−1^ indicates C = O stretching from carbonyl, carboxylic, and amide groups (Mendes et al. 2020), while absorption in the region 1600–1500 cm^−1^ can be assigned to aromatic C = C stretching. Additional contributions in the range 1200–1000 cm^−1^ are consistent with C–O stretching from alcohols, ethers, and polysaccharides, which are abundant in lignocellulosic biomass (Apinyakul et al. 2024).

In addition, characteristic signals of proteinaceous matter can be observed. The absorption bands in the range of 1650–1630 cm^−1^ arise mainly from C = O stretching vibrations of peptide bonds, while the band near 1550–1530 cm^−1^ is associated with N–H bending coupled to C–N stretching. A weaker contribution in the 3300–3200 cm^−1^ region may also be related to N–H stretching of amines and amides. These spectral features indicate the presence of proteins naturally occurring in the malt bagasse.

After carbonization (red spectrum), the spectrum exhibits a significant decrease in the intensity of functional groups. The disappearance or reduction of the C = O stretching band at 1730 cm^−1^, the attenuation of O–H vibrations, and the marked decrease of amide bands demonstrate thermal decomposition and devolatilization processes, with partial removal of lignin-, cellulose-, and protein-derived functionalities (Fan et al. 2021). The material becomes more graphitized, retaining mainly aromatic structures, as suggested by the residual bands in the region of 1600 cm^−1^ (Tian et al. 2020; Ge et al. 2023).

For the AC_op_ (blue spectrum), the spectrum is almost featureless, with very weak or broad signals. This is typical for highly carbonized porous carbons, in which the surface is dominated by graphitic domains with few remaining heteroatom-containing groups (Sevilla and Fuertes 2009). The low-intensity band near 1570–1600 cm^−1^ may correspond to skeletal vibrations of aromatic C = C or conjugated C = O species, but the amide-related signals have completely disappeared, confirming the removal of proteinaceous and cellulosic matter during the activation process. Overall, the chemical surface becomes largely depleted of oxygenated and nitrogenated functionalities (Jais et al. 2021; Zhou et al. 2022).

In summary, the FTIR-ATR analysis demonstrated the progressive removal of hydroxyl, aliphatic, carbonyl, and protein-derived functionalities during carbonization and activation, reflecting the structural evolution from a functionalized lignocellulosic precursor (malt bagasse) to a highly aromatic, heteroatom-poor activated carbon material suitable for adsorption.

### Raman spectroscopy

**Fig. 7:**
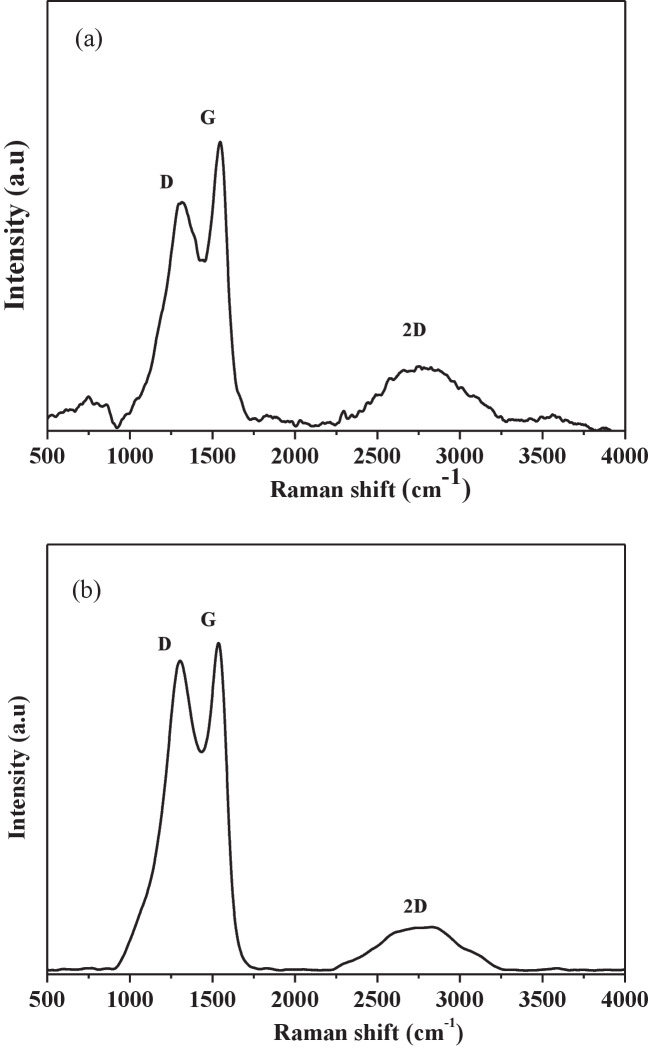
Raman spectra of **a** carbonized material and **b** AC_op_

The Raman spectra presented in Fig. 7 provide important insights into the structure of carbonaceous materials (Zhang et al. 2024). These spectra can be divided into two distinct regions: the primary bands region located between 1000 and 1700 cm⁻^1^ and the secondary band region, located between 2300 and 3300 cm⁻^1^ (Skrzypek 2021). The D band (around 1360 cm⁻^1^) and the G band (approximately 1560 cm⁻^1^) are observed in the primary region. The D band is associated with the breathing mode of carbon atoms in aromatic rings, becoming Raman active when symmetry is broken, while the G band corresponds to the stretching vibrations of C = C bonds in well-ordered sp^2^-hybridized domains (Ferrari and Robertson 2000; Maslova et al. 2021). The intensity ratio of these bands *I*_D_/*I*_G_ is a fundamental parameter for structural analysis, commonly used as an indicator of defect density, and is inversely related to the crystallite size (*L*_a_), as described by the Tuinstra and Koenig (1970) model. Thus, as the number of defects increases, the *I*_D_/*I*_G_ ratio rises (Li et al. 2023).

The quantitative values of the *I*_D_/*I*_G_ ratio and the average crystallite size (*L*_a_) are presented in Table S3. For the carbonized material, the *I*_D_/*I*_G_ ratio was 0.95, and the average crystallite size was 19.96 nm. In contrast, the optimized activated carbon (AC_op_) exhibited a higher *I*_D_/*I*_G_ ratio of 1.02 and a smaller L_a_ value of 18.65 nm, indicating the introduction of structural defects during the activation process. Thus, the carbonized material presents lower defect density and, consequently, a larger average size of graphitic crystallites (Orlando et al. 2021). In contrast, the spectrum of AC_op_, which reveals greater structural disorder due to the introduction of defects resulting from chemical activation with NaOH, reduces the crystallite size.

### CO_2_ adsorption studies

#### Adsorption isotherms

**Fig. 8:**
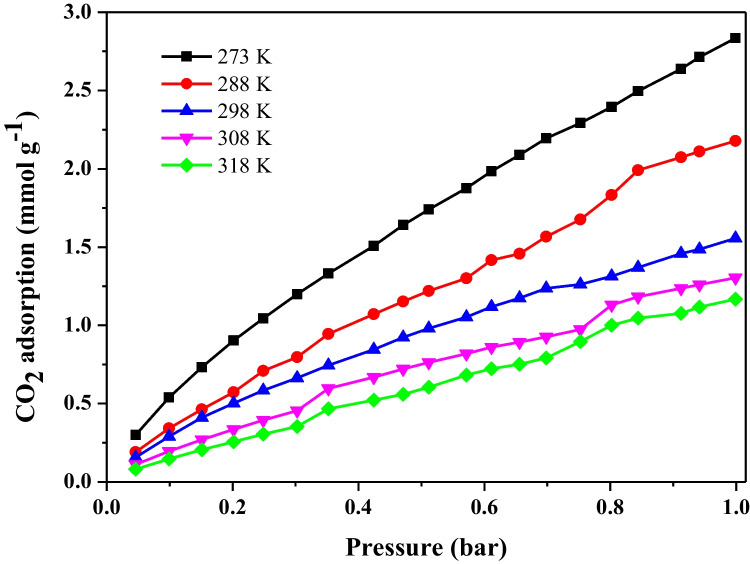
CO_2_ adsorption isotherms of AC_op_ at different temperatures

Figure 8 presents the CO₂ adsorption equilibrium isotherms obtained at temperatures from 273 to 318 K. According to the results, the amount of adsorbed CO₂ tends to decrease with increasing temperature, following the principle that the adsorption of gases on solid surfaces is always an exothermic and enthalpy-driven process (Adamson and Gast 1967). The isotherms exhibited a concave shape relative to the pressure axis across the entire investigated pressure range, which might be attributed to the monolayer adsorption (Thommes et al. 2015; Orlando et al. 2021). The data show that, at 273 K and 298 K and fixed pressure of 1.0 bar, the maximum adsorption capacity (q_e_) for AC_op_ was 2.83 and 1.56 mmol g⁻^1^, respectively. These temperatures and pressure were specifically selected to enable direct comparison with values commonly reported in the literature, as shown in Table 3. The results indicate that AC_op_ presents a CO_2_ adsorption capacity comparable to other materials previously reported.

**Table 3:** CO_2_ adsorption capacity on AC_op_ from malt bagasse is comparable to carbon-based materials reported in literature

Precursor	*S*_BET_ (m^2^/g)	*q*_e_ (mmol/g) at 1 bar	Adsorption temperature (°C)	Reference
Malt bagasse	884	2.83 (1.56)	0 (25)	This work
Avocado Seeds	700	2.37	0	(Siemak et al. 2024)
Bamboo-based	1492	1.45	25	(Ismail et al. 2022)
Argan paste	2046	4.83	0	(Yahia et al. 2024)
Pigskin	2799	2.20	25	(Gao et al. 2018)

#### Selectivity

Ideal adsorption solution theory (IAST) was used to calculate the selectivity of gas mixtures from the isotherms of pure gases. The selectivity of these materials plays a crucial role in a wide range of applications, including gas purification, molecular separation, and carbon capture. The adsorption of N_2_ and CO_2_ for AC_op_ at different temperatures from 273 to 318 K is shown in Fig. S3. The CO_2_ adsorption capacity was more significant than N_2_ at all temperatures and partial pressures.

**Fig. 9:**
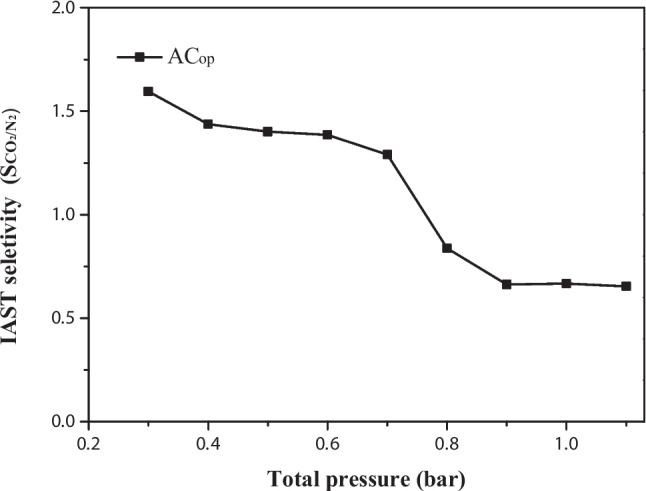
S_CO2/N2_ at 273 K for AC_op_ in a composition of 15% CO_2_ and 85% N_2_ in the total pressure range of 0.3 to 1.0 bar

Figure 9 shows the ${\mathbf{S}}_{{\mathbf{C}\mathbf{O}}_{2}/{\mathbf{N}}_{2}}$ at 273 K for AC_op_ with a gas composition of 15% CO_2_ and 85% N_2_, reflecting typical gas mixing ratios reported in the literature. The selectivity coefficient of CO_2_ relative to N_2_ varies from 1.7 to 0.6 as the pressure increases from 0.3 to 1.0 bar. At lower pressures, CO_2_ demonstrates a strong tendency to adsorb over N_2_, which is attributed to its larger molecular size and greater polarizability, leading to stronger interactions with the adsorbent surface (Zhang et al. 2021). However, as pressure rises, the selectivity coefficient declines, indicating a diminished adsorption preference of CO_2_ compared to N_2_. This decline may stem from a reduction in the number of sites available for strong CO_2_ adsorption over N_2_ (Hamyali et al. 2022). Nevertheless, the estimates have limitations at higher pressure values since IAST does not account for factors like pore saturation and gas–gas interactions, which could skew results away from ideal behavior (Adamson and Gast 1967).

#### Isosteric heat of adsorption

**Fig. 10:**
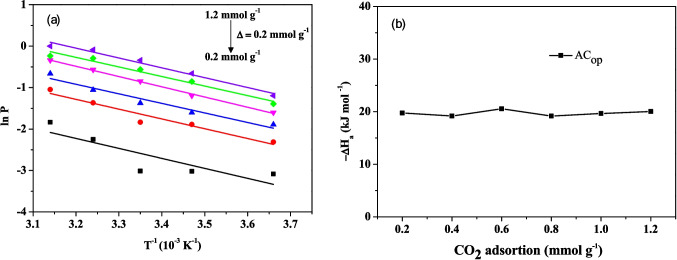
**a** ln P as a function of T^−1^ for different values of surface coverage (θ) and **b** isosteric heat of adsorption for AC_op_

Figure 10a represents the behavior of $\mathbf{ln}{\boldsymbol{P}}$ as a function of T^−1^ for different values of surface coverage $({\boldsymbol{\uptheta}})$. The graph shows that relative pressure increases with increasing coverage. Figure 10b relates the molar enthalpy change to the amount of CO₂ adsorbed, showing a nearly constant value of around 20 kJ mol⁻^1^, with only minor variations as adsorption progresses. This trend indicates that the AC_op_ surface is energetically homogeneous, with active sites of similar affinity for CO₂, and that lateral interactions between adsorbed molecules are minimal.

### High-pressure adsorption isotherms

**Fig. 11:**
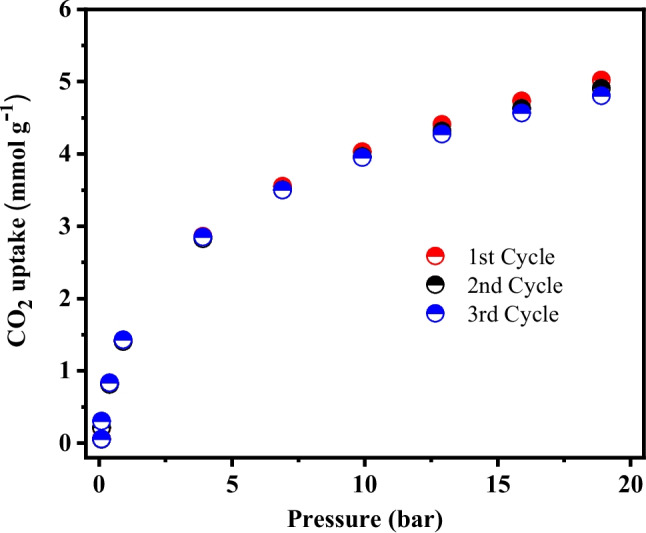
High-pressure CO_2_ adsorption isotherms at 25 °C of AC_op_ over consecutive adsorption cycles

Figure 11 presents the high-pressure CO_2_ adsorption isotherms measured at 25 °C over consecutive adsorption cycles. As shown, the material exhibits a high CO_2_ uptake under pressurized conditions, reaching 5.02 mmol g^−1^ in the first cycle, 4.90 mmol g^−1^ in the second cycle, and 4.80 mmol g^−1^ in the third cycle at approximately 20 bar. A small capacity loss (a.c. 2–4%) over consecutive cycles is observed, indicating excellent adsorption reversibility and confirming the structural stability of the material under repeated high-pressure operation. Overall, these results demonstrate that the material is well suited for cyclic adsorption technologies, particularly pressure swing adsorption, where high adsorption capacity at elevated pressures, efficient regeneration, and long-term stability are essential for practical applications.

## Conclusion

This study demonstrates that NaOH is capable of effectively developing a well-connected microporous network and significantly enhancing the surface area of carbons derived from malt bagasse. Binary mixtures $$(x_1x_2$$ and $$x_1x_2)$$ and the ternary mixture ($$x_1x_3(x_1-x_3))$$ led to a reduction in *S*_BET_, which can be attributed to competing reactions and partial pore blockage caused by secondary phases formed during activation. These results highlight the critical role of reagent selection in controlling pore formation, surface chemistry, and, ultimately, adsorption performance. By combining physicochemical analysis, statistical modeling, and a clear correlation between activation chemistry and textural properties, this work provides a robust framework for understanding and optimizing the activation of industrial residues. The findings not only reinforce NaOH as a highly effective activating agent but also offer fundamental insights that can guide the design of tailored porous carbons for CO₂ capture, demonstrating adsorption capacities of 2.83 mmol g^−1^ at 273 K. The analysis of the materials revealed that malt bagasse, a renewable and cost-effective raw material, can be effectively utilized in the production of efficient adsorbents, thereby promoting a sustainable approach to developing functional materials.

## Supplementary Information

Below is the link to the electronic supplementary material.

ESM1 (PDF 362 KB)

## Data Availability

Data will be made available on request.
