# Supplementary material for: Optimization of chemically activated carbon derived from malt bagasse for CO₂ adsorption: a simplex-centroid approach
Source: Environ Sci Pollut Res Int. 2026 Feb 27;33(9):3962–81. doi: 10.1007/s11356-026-37557-7 (PMC13043594; doi:10.1007/s11356-026-37557-7)
Supplement: Supplementary file 1 — (PDF 362 KB) [file 11356_2026_37557_MOESM1_ESM.pdf]

Supplementary material for

Optimization of chemically activated carbon derived from malt bagasse for CO_2 adsorption: a simplex centroid approach

Giovanna C. Carlos[1], Lucas H. S. Crespo[1], Pedro H. C. Voloch[1], Aline A. R. Andrade[1], Pedro H. V. Ribeiro[1], Faissal Robbin[1], Vitor C. Almeida[1], and Lucas Spessato[1*].

[1]*Department of Chemistry, State University of Maringá, Colombo Av. 5790, Maringá, Paraná, Brazil.*

Corresponding author:

* Tel: + 55 44 3011 3657. E-mail: lspessato@uem.br (Lucas Spessato)

Table S1. Model summary statistics.

Source	Std. Dev. (10^{-8})	R^2	Adjusted R^2
**Linear**	9.15	0.2438	0.1780
**Quadratic**	8.05	0.4908	0.3635
**Special Cubic**	8.24	0.4936	0.3337
**Cubic**	6.57	0.7285	0.5759

Table S2. Analysis of variance (ANOVA).

Terms	Quadratic Sum	Degrees of freedom	Quadratic mean	F-value	p-value
**Model**	$5.04x10^5$	9	55963.07	4.86	0.0030
**Linear mixture**	$1.69x10^5$	2	84535.66	7.35	0.0054
x_1x_2	$1.17x10^5$	1	$1.17x10^5$	10.15	0.0058
x_1x_3	73641.12	1	73641.12	6.40	0.0223
x_2x_3	1438.45	1	1438.45	0.13	0.7283
$x_1x_2x_3$	1952.80	1	1952.80	0.17	0.6858
$x_1x_2(x_1 - x_2)$	3446.46	1	3446.46	0.30	0.5917
$x_1x_3(x_1 - x_3)$	$1.22x10^5$	1	$1.22x10^5$	10.60	0.0050
$x_2x_3(x_2 - x_3)$	18229.00	1	18229.00	1.58	0.2262
**Residual**	$1.84x10^5$	16	11504.74		
**Total**	$6.87x10^5$	25			
**CV (%)**	24.7				

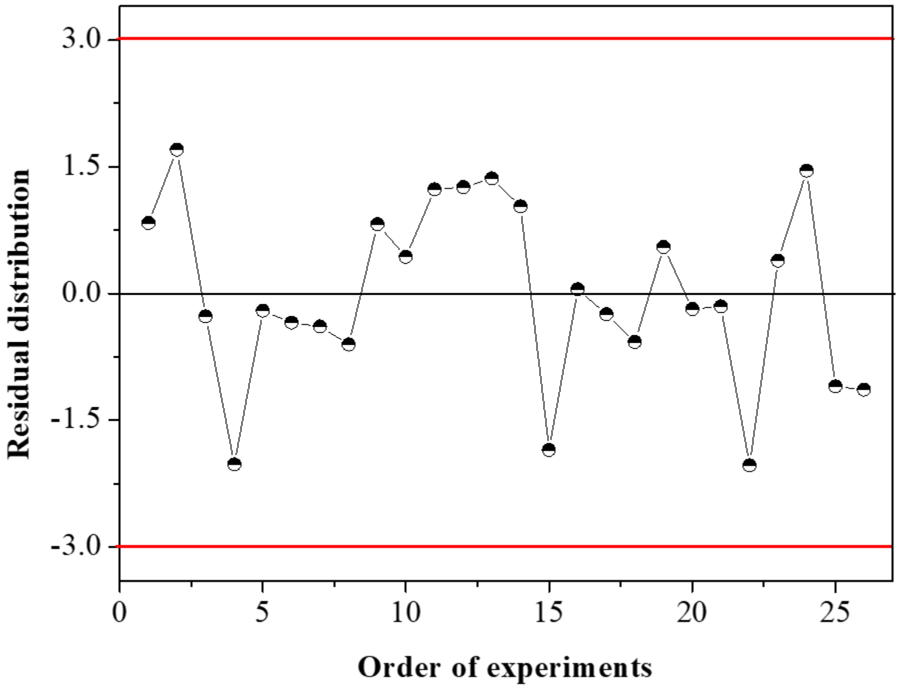

Figure S1. Plot of residuals versus order of experiments.

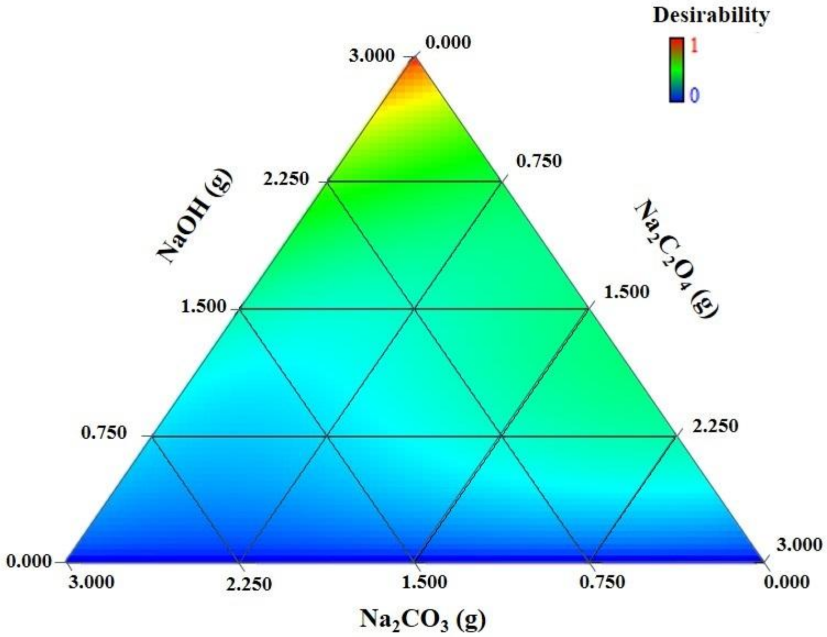

Figure S2. Desirability plot.

Table S3. I_D/I_G and L_a of the carbonized material and of the AC_{op}.

	**Carbonized material**	**AC_{op}**
$\boldsymbol{I_D/I_G}$	0.95	1.02
$\boldsymbol{L_a}$*(nm)*	19.96	18.65

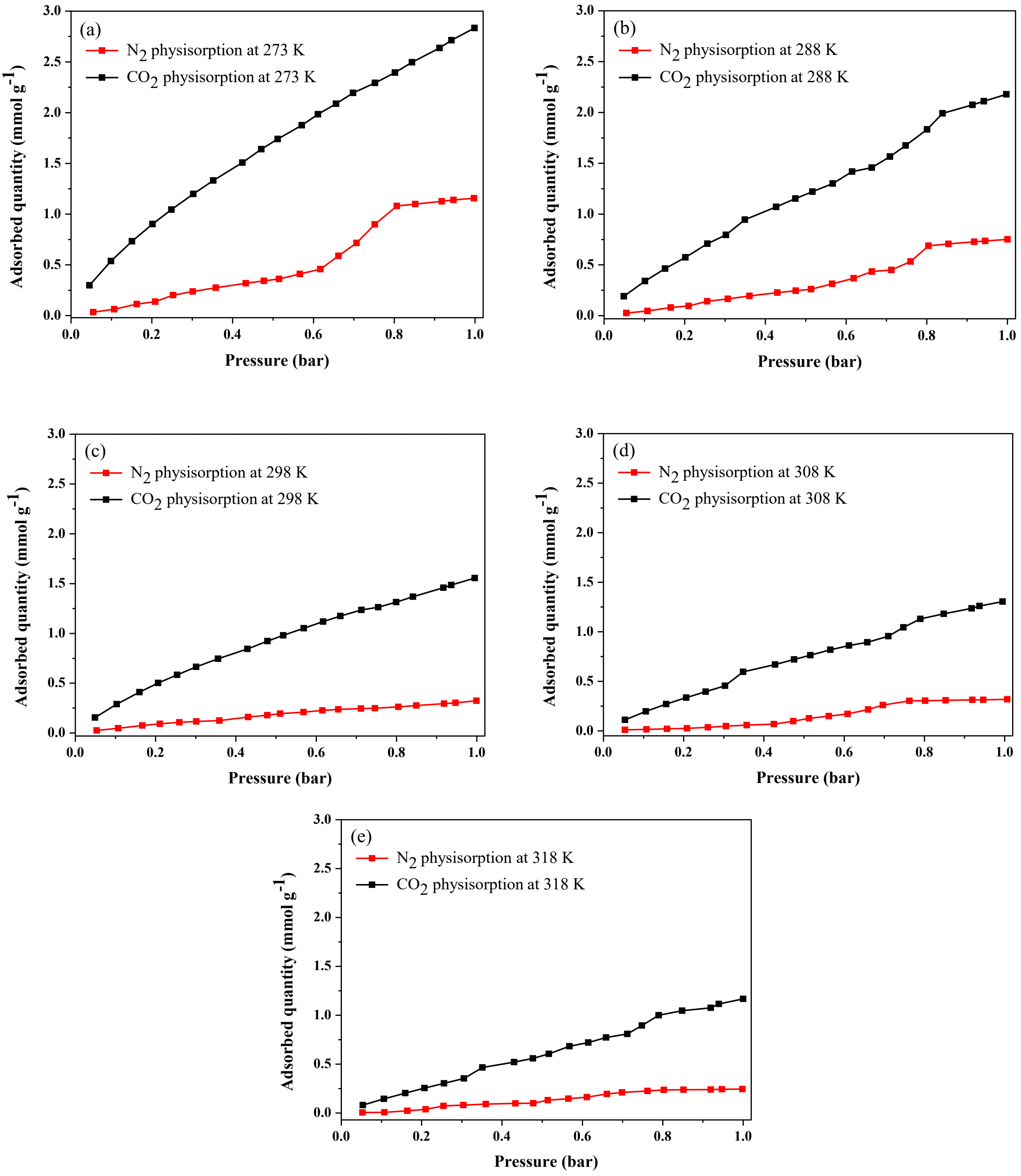

Figure S3. N_2 and CO_2 adsorption isotherms for AC_{op} at temperatures (a) 273 K, (b) 288 K, (c) 298 K, (d) 308 K, (e) 318 K.
